# A qualitative analysis of free text comments of participants from a massive open online mindfulness course

**DOI:** 10.3389/fpubh.2022.947898

**Published:** 2022-08-12

**Authors:** Sandra L. Neate, Jeanette C. Reece, Craig Hassed, Richard Chambers, Sherelle Connaughton, Nupur Nag

**Affiliations:** ^1^Neuroepidemiology Unit, Centre for Epidemiology and Biostatistics, Melbourne School of Population and Global Health, The University of Melbourne, Melbourne, VIC, Australia; ^2^Monash Centre for Consciousness and Contemplative Studies, Monash University, Melbourne, VIC, Australia

**Keywords:** mindfulness, qualitative analysis, digital intervention, mental health, knowledge translation

## Abstract

**Introduction:**

Mindfulness-based interventions are associated with improved health and wellbeing. Online mindfulness interventions offer potential scalability and cost advantages over face-to-face interventions. However, little is known about the experiences of learners, what they identify as being helpful to developing a practice of mindfulness and what outcomes they experience from undertaking an online mindfulness program.

**Methods:**

The Mindfulness for Wellbeing and Peak Performance Massive Open Online Course is a 4-week mindfulness program which includes psychoeducation, mindfulness meditation, applications and moderated discussion forums. Of the 3,335 participants who completed the March 2020 course, 527 (16%) responded to the final forum which invited participants to describe the highlights of the course. In order to enhance understanding of participant experiences and perceived outcomes of undertaking the course, a qualitative analysis of these free text comments was conducted using reflexive thematic analysis.

**Results:**

Two overarching themes were identified: (1) internal mechanisms of developing mindfulness (subthemes: paying attention to the present moment, learning to let go and find acceptance, cultivating an attitude of gentleness, and learning through a sense of belonging) and (2) the translation of mindfulness into daily living (subthemes: mindfulness being a support to mental wellbeing, learning to deal with uncertainty and adversity, living a more conscious life, a greater connection with self and others and channeling attention into productivity).

**Discussion:**

The themes and subthemes provided insights into the mechanisms learners used to develop mindfulness and how they translated mindfulness into their lives in a variety of beneficial ways. This understanding of learners' experiences could inform delivery of future online mindfulness interventions.

## Introduction

Mindfulness can be defined as the intentional direction of attention toward one's immediate experiences while adopting an open and accepting attitude (1). Being mindful is associated with improved mental health and wellbeing (2–4) including lower levels of stress, distress, depression and anxiety (5), and physical health benefits including reduced risk of cardiovascular disease (6). Mindfulness-based interventions (MBIs) aim to enhance participants' trait mindfulness and their ability to apply mindfulness to various aspects of everyday life and may also be used as therapeutic interventions in a range of mental and physical health conditions (7). Meta-analyses of the outcomes of MBIs demonstrate enhanced coping with distress (8), significant reductions in stress (9–11), anxiety (10) and depression (11, 12) and improved empathy and self-compassion (9). Mindfulness training may also be useful in programs such as smoking cessation (13), weight loss (14) and in occupational settings to reduce stress, burnout and improve job satisfaction (15, 16).

The increase in demand for self-help courses and online learning has led to online platforms becoming increasingly popular. Online interventions offer many potential advantages over face-to-face delivery such as accessibility, the ability to work in one's own environment, cost, anonymity and the potential lack of need for the presence of a therapist (17). They may also improve learning and program retention as learners are able to work at their own pace (17). For many people online MBIs are simply the preferred mode of delivery (18). Online MBIs have been compared with face-to-face interventions in randomized controlled trials and found to have similar outcomes (19). A large meta-analysis of the mental health outcomes of online MBIs found a small but significant improvement in depression, anxiety, wellbeing and mindfulness and a moderate effect for stress (7).

Massive open online courses (MOOCs) now provide courses to a large number of international learners, enabling access to information to those who have not previously had flexible, free, current content available (20, 21). However, there is little research on MOOCs which deliver mindfulness programs. Examination of existing MOOC literature revealed a systematic review of MOOCs from 2014 to 2016. This review identified that most studies focused on how learners engaged with the course materials, such as course completion, retention, attrition, while only 14% examined content and outcomes (22). Other reviews found that study methodologies tended to limit understanding of learner experiences by their dependence on quantitative data, or analytics drawn from usage patterns of course participants (23, 24). Researchers have favored a quantitative approach in MOOC research, preferring the collection of data via surveys and automated methods (24), with only 30% using qualitative methods alone and 5.5% adopting a thematic analysis approach (22). In order to generate a greater understanding of learner experiences and activities in MOOCs, more diverse research methodologies need to be employed (23). The combination of limited study numbers, the focus on topics such as completion rates and frequent use of quantitative methods, limits a more in-depth understanding of learner experiences and outcomes of mindfulness based MOOCs.

The Mindfulness for Wellbeing and Peak Performance (MWPP) MOOC (25) aims to teach mindfulness techniques to reduce stress and improve wellbeing and work/study performance. A quantitative evaluation of MWPP found that participation was associated with increased mindfulness, reduced perceived stress, and improved work engagement (26). The current study's significance is due to its use of qualitative methodology, uncommonly used in the MOOC literature, to add rich data to the findings about the MWPP and the MOOC literature in general. The study also explores infrequently examined topics, such as the mechanisms of developing mindfulness and incorporation into daily life of learned skills. To compliment previous quantitative data regarding the MWPP, we aimed to use a qualitative methodology to explore potential mechanisms mediating the development of mindfulness practice in MWPP participants and specific outcomes learners perceived.

## Methods

This study was approved by the Monash University Human Research Ethics Committee (ID: 18105) and the University of Melbourne Human Research Ethics Committee (ID: 14514).

### The massive open online course

MWPP (25) was developed in 2015 by co-authors CH and RC from Monash University, Australia, and its development and delivery have been described in detail elsewhere (26). MWPP was delivered over four weeks *via* FutureLearn, a United Kingdom digital learning platform, although participants were free to complete the course at their own pace, within a 6 week window. Content consisted of evidence underpinning mindfulness, the practice of mindfulness meditation, and the translation of mindfulness into daily living. Content was delivered *via* text, videos, links to relevant research and other articles, downloadable guided meditations, quizzes and discussion forums. Forums after each section of the course were moderated by course mentors who facilitated discussion, supported learners and addressed questions. At the end of each week's content, course educators responded to the main themes arising in forums *via* the recording of a weekly feedback video.

### Participants

Recruitment to the course was participant driven and enrolment was free of charge. Basic demographic data were collected and consent for use of anonymous data for research was obtained. In the 16th run of MWPP, delivered from March to April 2020, 23,932 people enrolled, 18,080 commenced the course and 3,335 (18%) completed at least 90% of modules. Of these 3,335 learners, 527 (16%) responded to the final discussion forum which included the question “What are the gems for you from this course?” Comments in languages other than English (13) and responses from mentors to learners (41) were excluded leaving 473 (14%) comments for analysis. All de-identified data was downloaded directly from the FutureLearn platform, assigned a unique author identification number, and imported into Microsoft Excel and then stored on a secure university server. As the data was received de-identified, participant demographics could not be reported.

### Data analysis

Within a qualitative paradigm, free text comments were analyzed using reflexive thematic analysis (27), an approach that facilitates the identification and analysis of patterns or themes in a dataset that is not tied to a particular philosophical approach (28). An inductive approach to analysis was applied whereby coding and theme development were derived from the raw data (29).

Two researchers repeatedly read all free text comments and independently coded the data. Codes fell into two broad categories: components of the course that assisted course completion and implementation of mindfulness; and mechanisms by which learners developed mindfulness and outcomes they reported. This study analyzed data from the second category. Codes from this second category were revised and renamed during many “sweeps” of the data until the researchers believed the codes accurately represented the participants' comments. Broader patterns of meaning were identified and initial themes generated. The data were collated under proposed themes and reviewed repeatedly to ensure the themes reflected the data and contained a central organizing concept. Memos of researcher meetings recorded theme development and researcher reflections (30). Verbatim data extracts were chosen to illustrate themes and enhance the transparency of the analysis (31).

## Results

Two themes (each with a number of subthemes) were identified from the data related to mechanisms learnt to practice mindfulness and the translating of mindfulness into daily practice.

Theme 1: Internal mechanisms of developing mindfulness

a) Paying attention to the present moment
b) Learning to let go and find acceptance
c) Cultivating an attitude of gentleness
d) Learning through a sense of belonging to the community

Theme 2: Translation of mindfulness into daily living

a) Mindfulness being a support to mental wellbeing
b) Learning to deal with uncertainty and adversity
c) Living a more conscious life
d) A greater connection with self and others
e) Channeling attention into productivity

### Internal mechanisms of developing mindfulness

#### Paying attention to the present moment

Many participants found that the first step in developing mindfulness was to focus their attention on the present moment. Learners noticed that their thoughts wandered and became aware of how they commonly responded to wandering thoughts. Practicing non-reaction and conscious redirection of their attention to the present moment helped avoid reliving past problems or concerns about the future.

*I spend most of my time living in the past or the future with everything that comes with that – regrets and worries. This 4-week course has helped me, or reminds me, to live in the present. (ID 505)*

The ability to direct attention to their senses improved awareness and appreciation of immediate surroundings and everyday experiences.

*How to sense and enjoy all small but constructive things and moments in life. (ID 42)*

Learners recognized that their thoughts were often not in the present moment and prompted them to question how their minds were normally occupied.

*This is so helpful to me because it begs me to ask the question…what am I giving my attention to in the moments that make up my daily life? (ID 69)*

#### Learning to let go and find acceptance

Learners identified the importance of being present in the moment and observing their thoughts, but then allowing those thoughts to pass. They reflected that learning to let go helped them allow whatever situation they faced to unfold and to relinquish efforts to control the situation.

*Mindfulness will help me to sit back and let things play out rather than having to “control” the situation. (ID 257)*

Many recognized the importance of acceptance to restoring a sense of internal balance.

*I now realize that to balance body and soul I personally need to let go. (ID 133)*

Other brought their acceptance to their work environments.

*Then begin to calmly do whatever I can to meet the deadlines and if I make it that is great, and if I don't, that is OK too (ID 209)*

#### Cultivating an attitude of gentleness

Learners developed new attitudes toward themselves, their thoughts and emotions, and their efforts to practice mindfulness. With the encouragement of the course educators and mentors, they developed a nonjudgmental, gentle attitude to themselves.

*Acknowledgment and understanding that I need to be gentle and non-judgmental with myself and let my mind be. (ID 411)*

Many learners expressed frustration at their wandering minds and sometimes intrusive thoughts while practicing mindfulness. They felt adopting an attitude of gentleness assisted in the practice of mindfulness.

*Gently guiding thoughts back as many times as needed. This has taken some of the frustration and sense of failure away when thoughts wander. (ID 309)*

Learners realized that being non-judgmental toward themselves helped them to notice their thoughts and emotions and let them go.

*The biggest gem for me was that I can acknowledge an emotion, put it to one side and not be judgmental about it. Just let it be. (ID 131)*

The gentleness and non-judgmental attitudes were in contrast to previous tendencies for self-criticism.

*I recognize tendencies toward perfectionism but couldn't reconcile that with why I was so useless as a young student. Now, I realize that it was down to an intense fear of failure, so I essentially self-sabotaged. (ID 301)*

#### Learning through a sense of belonging to the community

The discussion forums were an important feature of the course that helped foster a sense of community amongst participants. Course mentors and other participants responded to learners' comments, answering questions, and providing support and encouragement. Shared experiences and the kindness of others in the online community were highly valued as a support to learning mindfulness.

*The amazing other learners who share so generously their experiences and kindness.…We all learn together. (ID 145)*

Learners commented on the value of the support, sense of belonging and the safe and comforting online environment. This was highly valued, especially as many struggled with the isolation and challenges of the global pandemic which was underway at the time.

*Really comforting. I'm glad there are other people struggling in some ways with the online course but still being able to go through it. I feel very proud of each person that has taken this course because it is definitely not easy to focus enough at home, especially during this time of stress and sadness for the global situation. (ID 354)*

### Translation of mindfulness skills into daily living

#### Mindfulness being a support to mental wellbeing

Many learners had undertaken the course to support and improve mental health and wellbeing, in the face of personal and collective life challenges. Learners felt that the skills they developed supported their mental health in many ways, providing the opportunity to pause and reflect.

*With everything that is happening around the world with this pandemic, our mental health can be easily challenged. Mindfulness can give us the time to stop and reflect and possibly help us in this difficult circumstance. (ID 404)*

Developing a mindfulness practice and living mindfully led to a sense of calmness and balance.

*It has very much helped me during this time whilst having to make big decisions and also maintaining a sense of calm and mental balance. (ID 22)*

Remaining in the present meant that some motivations for undertaking the course, including the reduction of stress and anxiety, were realized as part of redirecting attention back to the moment.

*I'm no longer consumed by anxiety as it's just noise in the background which I can choose to quieten or ignore. (ID 269)*

#### Learning to deal with uncertainty and adversity

Many learners had experienced greater uncertainty about the present and the future. The practice of mindfulness translated into ways of dealing with the challenges of pandemic related lockdowns and the inability to be certain or plan. Mindfulness had provided a sense of peace in the face of this uncertainty.

*How calm and content I have been during lockdown, doing what is necessary but also seeing “the silver lining” and feeling gratitude. (ID 286)*

Many learners had experienced adversity directly attributable to the pandemic and mindfulness had provided enhanced coping skills, some of which were applicable to handling varying aspects of life.

*A bit more than 4 weeks into quarantine, jobless and lost a few family members and I know this course helped me going through all of that. (ID 285)*

#### Living a more conscious life

Many wrote learned mechanisms of present moment attention and non-judgmental observation of thoughts and emotions resulted in living more mindfully. They recognized the ways they had reacted to emotions and were more able to observe with less reaction.

*Mindfully managing your emotions. That it's ok to have emotions and even so-called negative emotions but it's how you react to your emotions that really matter (ID 285)*

Learners were able to use the skills they had developed in practical situations, such as recognition of reacting in a mindless manner and responding impulsively to situations and were able to adjust these responses.

*I am becoming less reactive to situations that unfold and noticing them rather than impulsively reacting. (ID 502)*

Learners identified their entrenched patterns of thinking, that were they functioning in “default mode”, and how to return their attention in their daily lives to the present moment.

*I'm ending the course with a greater understanding of the mind's continual pull to a default stance and with strategies to keep bringing it back to the present. (ID 383)*

They became more able to mindfully interact with others and undertake daily activities.

*I feel ready to walk this way. Not just meditating, but mindful living. Aware of how I engage with my family and friends, my colleagues, the food I eat. (ID 32)*

#### A greater connection with self and others

Learners identified that they had developed enhanced awareness of and connection with themselves. This provided an opportunity to re-evaluate the way they had been living. They recognized they had been living life at a frantic pace and tried to slow down.

*With so many activities and the pace of life nowadays, it is always good to have time to be connected to yourself and to the present. (ID 451)*

Skills provided the ability to connect better with others *via* active listening and remaining attentive to the conversation.

*Listen carefully, without judgment, and not thinking of our own reply, whilst the other person is speaking. And when replying we reply with only what is necessary, not overstating or understating things. (ID 47)*

Connection with others included improved relationships and communication with family members and others in personal lives.

*It improved my relationship with my mother as I'm not so reactive as I used to be. (ID 265)*

And the connection, for some, extended to the wider community, the development of concern and compassion for others and a greater connection with nature and the environment.

*I remembered to especially enjoy the cycle ride-mindful of the potholes and noticing the tree blossom, daffodils, lambs in the fields and birdsong. (ID 87)*

#### Channeling attention into productivity

This subtheme explored how learners used the learned mechanisms of mindfulness to enhance their efficiency and productivity in many aspects of life. Remaining in the present meant better performance and improved ability to deal with competing demands. Learners reflected on the importance of channeling attention toward one thing at a time rather than multi-tasking, finding their efficiency was enhanced.

*But most useful of all will be the ability to quit multi-tasking and replace it with efficient time and attention switching. (ID 439)*

Others reflected on productivity in terms of enhanced creativity.

*I've learned how precious mindfulness is to creativity, I've listened to (the) creativity mindfulness meditation before embarking on my creative writing each day this week and its really brought me into the right space to write about topics including grief and loss. (ID 210)*

## Discussion

Teaching mindfulness *via* an online course represents a valuable low-cost tool, especially for those living with a disability, remotely or during extreme situations such as a pandemic or war. However, few studies have qualitatively examined participants' experiences with massive online mindfulness courses, in particular, how they learn mindfulness from online courses, benefits they perceive from mindfulness and the translation of what they learn into their daily lives. Our qualitative study used thematic analysis to explore participants' experience of MWPP, a popular online mindfulness course, and two overarching themes were identified: (1) internal mechanisms of developing mindfulness and (2) the translation of mindfulness into daily living.

Our data provided validation of the first theme, and subthemes demonstrated that participants gained a unified and collective set of interacting mindfulness mechanisms, as opposed to a single mechanism. Notably, this combination of mechanisms has been found to be imperative for the development of mindfulness (32). Moreover, the mechanisms identified are consistent with previously reported interactive qualities of mindfulness practice which involve a process of inquiry that assist learners to identify their thoughts, emotions, and sensations, recognize habitual patterns of reacting to them, and respond with greater awareness and flexibility (33). This inquiring, educative philosophy had been built into MWPP from the outset. Several identified mechanisms echoed Kabat-Zinn's seven pillars of mindfulness (34). Developing a non-judgmental attitude, acceptance and letting go were explicitly discussed within course content, whereas other pillars were implicit within the content.

The subtheme of “paying attention to the present moment” within the theme of mechanisms of developing mindfulness, concurs with much of the mindfulness literature relating to the “hows” of mindfulness. Kabat-Zinn (35) described mindfulness as paying attention on purpose in the present moment. Several other studies have recognized monitoring present-moment experiences with an orientation of acceptance as a predominant attitude for mindfulness practice (1, 36). Baer and colleagues (37) summarized the “whys and hows” of mindfulness as “paying attention to the present moment with qualities of openness, non-judgment, acceptance, friendliness, curiosity, kindness, and compassion” and our findings of paying attention, acceptance, and cultivating an attitude of gentleness, are aligned with these. In a randomized controlled trial of 118 participants conducted in the United Kingdom, the effectiveness of an MBI was attributable to increased levels of non-judging (38), a commonly identified mechanism of developing mindfulness. However, curiosity is an additional reported quality previously associated with being in the moment (1) that was not identified in our qualitative analysis.

The subtheme “learning through a sense of belonging to the community” has also been identified as a mechanism of enhanced learning in people coping with life-changing chronic conditions. In a meta-analysis of six qualitative studies examining MBIs in people with multiple sclerosis “a sense of belonging” was identified as one of the four major themes with shared (mindful) learning identified as a vital mechanism (39). Similarly, stroke survivors (40) and people with amyotrophic lateral sclerosis (41) found the shared experience of MBIs with their peers provided motivation and support. The “sense of belonging” subtheme highlighted that the feeling of connectedness with other group members was an important mechanism of learning for some people.

The second theme, “translation of mindfulness into daily living”, explored learners' experiences of changes to their lives resulting from the internal mindfulness mechanisms acquired. Within this theme, the subtheme of mindfulness as a support to mental wellbeing is consistent with previously reported studies examining outcomes following MBIs and a very positive finding. MBIs conducted both before and during the pandemic (42) were found to be effective at reducing depression (43), perceived stress and anxiety (38, 43–45) and improving wellbeing and emotional regulation (44), with similar effectiveness to face-to-face programs (38, 46). Notably, previous studies have also documented some potential negative effects of MBIs including exacerbation of anxiety, depression and low self-esteem (33), with one study reporting participants felt overwhelmed by awareness of prior maladaptive coping habits, that present-moment awareness was occasionally frightening and that some had mistaken expectations regarding outcomes (47). As this study focused on a question that explored the gems of the course, learners did not report adverse events within the comments analyzed. “Pastoral care” for online learners was a key design consideration of the MWPP. The potential for adverse events was discussed in the introductory videos, learners were advised to go at their own pace, only practice what they were comfortable to practice, and to stop if they had any significant difficulties and to seek advice. These messages were reinforced in the moderation of the discussion forums and the feedback videos in response to learners' questions and comments. A gentle, careful and caring approach was maintained throughout the course to minimize adverse events.

The subthemes “dealing with uncertainty and adversity” and “living more consciously” have previously been recognized as fundamental benefits of MBIs (48–50). Outcomes of an online MBI conducted during the COVID-19 pandemic, included reduced difficulties in emotional regulation and increased tolerance to uncertainty (44). In our study, participants described, as part of “living more consciously”, greater understanding of habitual ways of responding and the ability to better recognize and regulate their emotions and responses. This concept has been described “as a reduced influence of habitual verbal-conceptual processes on the interpretation of ongoing experience” (51) described by Desbordes and colleagues as an attitude as equanimity (52). In our study, learners expressed the ability to regulate responses and emotions were translated into improved personal and work-related relationships and other benefits.

The subtheme of “connecting with self and others” explored how learners improved their understanding of themselves and others and experienced improved relationships. Improved relationships with others were further enhanced by having learned to regulate emotions and through non-judgmental listening and understanding. Monitoring present-moment experiences with an orientation toward acceptance has been found to change the way people perceive and relate toward others (37). Other studies have also reported improved social connectedness resulting from MBIs. Participants in an MBI using smart phones that included interaction with the course educators, reported increased connectedness and reduced loneliness and social isolation (36). People with multiple sclerosis undertaking an MBI experienced feelings of camaraderie and belonging (39). Consistent with our study, in Buddhist contemplative training, self-control practices incorporating acceptance nurture an attitude of compassion toward oneself and others, enhancing social connectivity (52).

In the subtheme of “channeling attention into productivity”, participants found focusing on the present task, regulating responses to deadlines, unitasking and efficiently switching attention, all improved their productivity. Other studies, including a prior study of the MWPP, recognized improvements that mindfulness can bring to productivity (26). A workplace MBI improved team and organizational “climate” and individual performance with greatest improvements seen in co-operation between team members (53). Additionally, a meta-analysis of workplace MBIs found these programs reduced perceived stress and health complaints, improved wellbeing and engagement, productivity and job satisfaction (16). Mindfulness was found to assist in creativity by our participants and limitations of our study are noted. Firstly, participants voluntarily enrolled in the MWPP thus making them more likely to be motivated and/or committed to mindfulness practice. Further, as the question examined asked participants to describe the “gems” of the course, this would likely have encouraged participants to only provide positive feedback. In addition, the study sample comprised a small fraction of the 18% of those who completed 90% or more of the modules. This sample had therefore invested heavily in the course compared with those with lower completion rates and were likely to view it more favorably, Our results may therefore not be generalizable to the general population or, indeed, all course participants. However, the non-directional open-ended question could be considered a strength of the study as participants were not prompted to provide directed answers but instead were likely to report the most relevant and memorable areas of the course. 473 (14%) of learners who completed the course were able to identify at least one “gem” of the course with 14% considered a high response rate for MOOC discussion forums. Further, as MWPP was specifically designed to improve peak performance (25) productivity related quotes may have been more pronounced than otherwise. Although pastoral care is provided within both content and facilitator feedback videos and *via* moderators of the forums, there is no explicit request for the reporting of adverse events and it is possible that unreported adverse events may exist. As the challenges learners experience, and the way they deal with these challenges, were not able to be considered, our findings may not fully explain how participants learn about mindfulness.

The study's impact lies within the finding that the MWPP provided learners with the recognized tools for developing mindfulness and that learners were able to identify those mechanisms. Developing an understanding of the mechanisms, in addition to the simple practice of mindfulness, may be of great value to the practice and implementation of mindfulness. The MWPP has previously been found to improve wellbeing, stress and peak performance (26) but employing a qualitative methodology has identified benefits beyond these. This qualitative exploration has identified the rich personal experiences and many positive outcomes of learners, such as mindfulness being a support for mental health and wellbeing. These promising results arising from the study indicate the MWPP could be a suitable toolkit for people to use in order to overcome psychological distress while also being mindful. Other important findings including an improved ability to deal with uncertainty and adversity, an increased connectedness with others and beneficial impact on relationships resulting from improved emotional regulation and living more mindfully.

Future directions include the inclusion of formal opportunities to discuss adverse events. Such information may assist in understanding challenges learners experience and how to best support learners. Views of those with lower completion rates could provide valuable insights regarding what, and if, anything should be changed and would also be a useful addition to this and other courses. These inclusions and the rich and detailed information gleaned from this study may inform future development of this and other MBIs.

Overall, our study provided evidence of the mechanisms by which learners developed mindfulness and the translation of these skills into their daily lives. Importantly, participants were able to identify how they developed these skills. The learners' awareness of these internal mechanisms assisted participants to apply these skills to everyday life with reported beneficial outcomes. Developers of future MBIs may wish to use the features employed in MWPP to assist in the effective delivery of an intervention that provides both the insight into the mechanisms of mindfulness and the ability to translate these skills into daily living.

## Data availability statement

The raw data supporting the conclusions of this article will be made available by the authors, without undue reservation.

## Ethics statement

This study was approved by the Monash University Human Research Ethics Committee (ID: 18105) and the University of Melbourne Human Research Ethics Committee (ID: 14514). The patients/participants provided their written informed consent to participate in this study.

## Author contributions

Conceptualisation: SN, NN, CH, and RC. Methodology, investigation, and formal analysis: SN and NN. Visualization, supervision, and project administration: SN. Resources: CH. Validation: CH, RC, and SC. Original draft preparation: SN and JR. Writing—review and editing: SN, JR, NN, CH, RC, and SC. All authors contributed to the article and approved the submitted version.

## Conflict of interest

CH and RC are creators and educators of the MWPP course and SC is a course mentor and moderates the discussion forums. The remaining authors declare that the research was conducted in the absence of any commercial or financial relationships that could be construed as a potential conflict of interest.

## Publisher's note

All claims expressed in this article are solely those of the authors and do not necessarily represent those of their affiliated organizations, or those of the publisher, the editors and the reviewers. Any product that may be evaluated in this article, or claim that may be made by its manufacturer, is not guaranteed or endorsed by the publisher.
